# The immunosuppressive activity of myeloid-derived suppressor cells in murine Paracoccidioidomycosis relies on Indoleamine 2,3-dioxygenase activity and Dectin-1 and TLRs signaling

**DOI:** 10.1038/s41598-023-39262-8

**Published:** 2023-07-31

**Authors:** Valéria de Lima Kaminski, Nycolas Willian Preite, Bruno Montanari Borges, Bianca Vieira dos Santos, Vera Lucia Garcia Calich, Flávio Vieira Loures

**Affiliations:** 1 Institute of Science and Technology, Federal University of São Paulo – UNIFESP, São José dos Campos, SP Brazil; 2 Department of Immunology, Institute of Biomedical Sciences, University of São Paulo – USP, São Paulo, Brazil

**Keywords:** Infection, Immunology, Fungal infection

## Abstract

Paracoccidioidomycosis (PCM) is a systemic mycosis with a high incidence in Latin America. Prior studies have demonstrated the significance of the enzyme Indoleamine 2,3-dioxygenase (IDO-1) in the immune regulation of PCM as well as the vital role of myeloid-derived suppressor cells (MDSCs) in moderating PCM severity. Additionally, Dectin-1 and Toll-Like Receptors (TLRs) signaling in cancer, infection, and autoimmune diseases have been shown to impact MDSC-IDO-1^+^ activity. To expand our understanding of MDSCs and the role of IDO-1 and pattern recognition receptors (PRRs) signaling in PCM, we generated MDSCs in vitro and administered an IDO-1 inhibitor before challenging the cells with *Paracoccidioides brasiliensis* yeasts. By co-culturing MDSCs with lymphocytes, we assessed T-cell proliferation to examine the influence of IDO-1 on MDSC activity. Moreover, we utilized specific antibodies and MDSCs from Dectin-1, TLR4, and TLR2 knockout mice to evaluate the effect of these PRRs on IDO-1 production by MDSCs. We confirmed the importance of these in vitro findings by assessing MDSC-IDO-1^+^ in the lungs of mice following the fungal infection. Taken together, our data show that IDO-1 expression by MDSCs is crucial for the control of T-cell proliferation, and the production of this enzyme is partially dependent on Dectin-1, TLR2, and TLR4 signaling during murine PCM.

## Introduction

Paracoccidioidomycosis (PCM) is a systemic, chronic mycosis endemic to Latin America caused by fungi of the genus *Paracoccidioides*. The infection results from the inhalation of mycelial fragments or conidia into the lungs, which can lead to a latent infection. However, the progression of the primary infection or reactivation of a latent focus can cause overt disease, which can be acute or chronic^1–6^.

The immunosuppression in PCM has been recently addressed. A preliminary investigation revealed that regulatory T-cells (Treg) have a deleterious effect on the disease in resistant (A/J) or susceptible (B10.A) mice with *P. brasiliensis* infection. The removal of Tregs resulted in a less severe and regressive infection in both mouse strains^7,8^. Further analysis of the inhibitory effect of Foxp3^+^ Tregs in pulmonary PCM was conducted by depleting Treg cells in DEREG (Diphtheria Toxin Receptor GFP transgenic) mice, which resulted in reduced fungal loads in multiple organs, decreased tissue damage, and lower mortality rates compared to control mice^9^. Importantly, the severity of human PCM has been linked to the suppressive activity of Foxp3^+^ Tregs, which appear in high numbers in the lesions and blood of severely affected patients^10,11^.

Myeloid-derived suppressor cells (MDSCs) are a heterogeneous population of immature cells that can impair immune responses. These cells include morphologically distinct subsets of monocyte-like MDSCs (M-MDSCs) and polymorphonuclear-like MDSCs (PMN-MDSCs) and are implicated in immune regulation during pregnancy and in diseases involving chronic inflammation, including infections, autoimmune diseases, and other pathologies^12–14^. Although few studies have addressed the involvement of MDSCs in fungal infections^15,16^, the role of MDSCs in PCM has been recently investigated by our group. Preite et al.^17^ showed that MDSCs are recruited to the lungs of *P. brasiliensis*-infected mice and express the immunosuppressive enzyme Indoleamine 2,3 Dioxigenase-1 (IDO-1), which contributes to impaired Th1 and Th17 responses in the disease, resulting in a severe pulmonary PCM that could be reversed by MDSC depletion in mice^17^.

The expression of IDO-1 is a major mechanism of immunosuppression in a variety of cancer types^18^, and the infiltration of MDSCs has been associated with this enzyme activity^19^. Furthermore, in different pathological contexts the expression of IDO-1 was already shown to be induced by pattern recognition receptors (PRRs), such as Dectin-1^20^, Toll-like receptor 2 (TLR2)^21^, and TLR4^22^. Moreover, these PPRs are involved in the activity or recruitment of MDSCs in several infectious diseases^15,23,24^. For instance, the induction of MDSCs after *Aspergillus fumigatus* and *Candida albicans* infections has been reported to be dependent on Dectin-1/CARD9 signaling, resulting in ROS generation and caspase-8 activity, along with IL-1β production^15^. The induction of inflammation via PRRs by innate immune cells requires careful control to avoid damage to host tissues. This regulation depends on intracellular receptors, membrane-bound suppressors, intracellular negative regulators, the degradation of TLRs, apoptosis induced by TLRs, and also the activity of MDSCs^23^. Intriguingly, MDSCs can be induced by TLR agonists, such as lipopolysaccharide (LPS), or whole pathogens^23^. In general, TLR2 and TLR4 cognates derived from microorganisms are capable of inducing monocytic MDSCs, as demonstrated in previous hepatitis C virus (HCV) and *Staphylococcus aureus* infection studies^24,25^. Specifically, TLR2 agonists, such as those present in *S. aureus,* can promote the differentiation of monocytes into MDSCs, leading to their accumulation in skin lesions^26,27^. In PCM, the involvement of Dectin-1, TLR2, and TLR4 has already been extensively studied^28–40^. However, their involvement in MDSCs recruitment and activity during pulmonary PCM has yet to be investigated. Thus, we aimed to evaluate the role of IDO-1 in MDSCs activity and the influence of Dectin-1, TLR2, and TLR4 on the production and activity of this immunosuppressive enzyme by MDSCs in a pulmonary model of *P. brasiliensis* infection in mice.

## Methods

### Mice

The experiments were performed in accordance with Brazilian Federal Law 11,794, as well as in accordance with the ARRIVE guidelines. The study was approved by the Ethics Committee on Animal Experiments of UNIFESP (Protocol Nº 2135170220). Eight- to 12-week-old male C57BL6/J WT, TLR2KO, and TLR4KO mice were bred as specific pathogen-free mice at the Center for the Development of Experimental Models for Biology and Medicine and were kept in the facility of the Institute of Science and Technology of UNIFESP. Also, eight- to 12-week-old male C57BL/6 Dectin-1KO, IDO-1KO, and WT mice were obtained from the specific pathogen-free isogenic breeding unit of the Department of Immunology, Institute of Biomedical Sciences, University of São Paulo.

### Fungus and infection

The virulent *P. brasiliensis* 18 (Pb18 isolate) yeast cells were cultured weekly in Fava Netto culture medium at 37 °C and used on days 7–8 of culture. The viability of yeasts, which was consistently higher than 95%, was determined using Janus Green B vital dye (Merck). Mice were anesthetized and subjected to intratracheal (it.) infection as previously described^41^. Briefly, after intraperitoneal (ip) injection of ketamine (90 mg/kg) and xylazine (10 mg/kg), animals were infected with 1 × 10^6^ yeast cells in 50 μL of phosphate-buffered saline.

### Flow cytometry

Cell suspensions were added to 96-well U-bottoms, and Fc receptors were blocked using 10 ng/mL Fc block (eBiosciences) for 10 min at 4 °C. Plates were washed twice with FACs Buffer (Biolegend). Subsequently, 25 μL of a mixture containing 1% of each fluorochrome-conjugated antibody used for MDSC identification was added and incubated for 25 min at 4 °C. The conjugated antibodies used for MDSC identification were Live/Dead-BV510, CD45-BV605, CD11b-APCCy7, Ly6C-APC R700, and Ly6G-BV421. The gating strategy for MDSC identification is shown in Suppl. Fig. 1A. We also evaluated the intracellular expression of IDO-1 in MDSCs using anti-mouse IDO-1 Alexa 647 (Biolegend). Thus, cells were treated with the fixation/permeabilization buffer (BD Biosciences) for 20 min at 4 °C. The cells were then washed and submitted to intracellular staining with a 25 μL mix of a FACs buffer containing 2% of the anti-IDO-1 antibody for 30 min at 4 °C (Suppl. Fig. 1B). T-lymphocytes were assessed using Live-Dead/BV510, anti- CD4/Alexa 647, anti-CD8-PECy7, anti-CD25-PE, and anti-CD69-APC Cy7 (Suppl. Fig. 2).

### In vitro generation of MDSCs

Bone-marrow-derived MDSCs (BM-MDSCs) were generated from naïve C57BL/6 WT, IDO-1KO, Dectin-1KO, TLR2KO, and TLR4KO mice as previously described^42^. All animals were euthanized with intraperitoneal (ip) injection of ketamine (270 mg/kg) and xylazine (30 mg/kg). BM cells were flushed out from the femurs and tibias of mice using a 1 mL syringe and Dulbecco's Modified Eagle Medium (DMEM, Sigma-Aldrich) supplemented with 3% fetal bovine serum. Red blood cells (RBC) were lysed in RBC lysis buffer (BioLegend) for 4 min. Cells were then washed with DMEM, and 7 × 10^5^ white blood cells per mL were cultured in cell culture bottles with DMEM supplemented with 10% FBS, recombinant murine IL-6, and granulocyte-monocyte colony-stimulating factor (GM-CSF), both at 10 ng/mL (Biolegend), and cultured for three days at 37 °C in a 5% CO_2_ chamber. BM-MDSCs were separated from other myeloid cell populations using the MDSC-isolation kit (Miltenyi), following the manufacturer’s instructions.

### Blockade of IDO-1, Dectin-1, TLR2, and TLR4

BM-MDSCs obtained from C57BL/6 WT animals were treated or not with 200 μM 1-methyl tryptophan (1MT, Sigma-Aldrich). Subsequently, 2 × 10^5^ BM-MDSCs were seeded per well in a 96-well U-bottom plate, treated or not with 1MT overnight, and then challenged with 4 × 10^3^ *P. brasiliensis* yeasts. In complementary experiments, BM-MDSCs were treated or not with 10 μg/mL of anti-Dectin-1 (Thermo Fisher), anti-TLR2, or anti-TLR4 (Invitrogen). A monoclonal IgG2b (BioxCell) was used as an isotype control.

### Immunosuppressive activity of BM-MDSCs on T-lymphocytes

We generated single-cell suspensions from the spleens of naïve C57BL/6 WT mice. After RBC lysis, T-cell populations were separated using the Pan T-Cell Isolation Kit (Miltenyi). To evaluate the proliferation of T-lymphocytes, cells were stained with carboxyfluorescein succinimidyl ester (CellTrace™ CFSE Cell Proliferation Kit, Invitrogen) according to the manufacturer's instructions. Subsequently, 1 × 10^6^ T-lymphocytes were activated with 1 µg/mL anti-CD3 and anti-CD28 monoclonal antibodies (BioLegend) and co-cultured at 37 °C and 5% CO_2_ for four days in the presence or absence of 1 × 10^5^ BM-MDSCs per well in a 96-well U-bottom plate. BM-MDSCs were previously challenged or not with 4 × 10^3^ *P. brasiliensis* yeasts. The proliferation of T-cells was defined according to the CFSE dilution and assessed by flow cytometry as outlined in the Suppl. Fig. 3^17,43^.

### Lung-infiltrating MDSCs in *P. brasiliensis*-infected mice

Lung tissues were collected from C57BL/6 WT, IDO-1KO, Dectin-1KO, TLR2KO, and TLR4KO *P. brasiliensis*-infected mice after 72 h, two weeks, and eight weeks of infection, respectively. The tissues were enzymatically digested in RPMI medium with 10% FBS containing 2 mg/mL of collagenase (Sigma-Aldrich) for 40 min at 37 °C and 120 rpm in a shaker incubator. Lung leukocyte suspensions were obtained as previously described^32^ and subjected to cell staining as described above.

## Results

### Treatment of MDSCs with an IDO-1 inhibitor (1-MT) decreased their suppressive activity on T-cells

**Figure 1:**
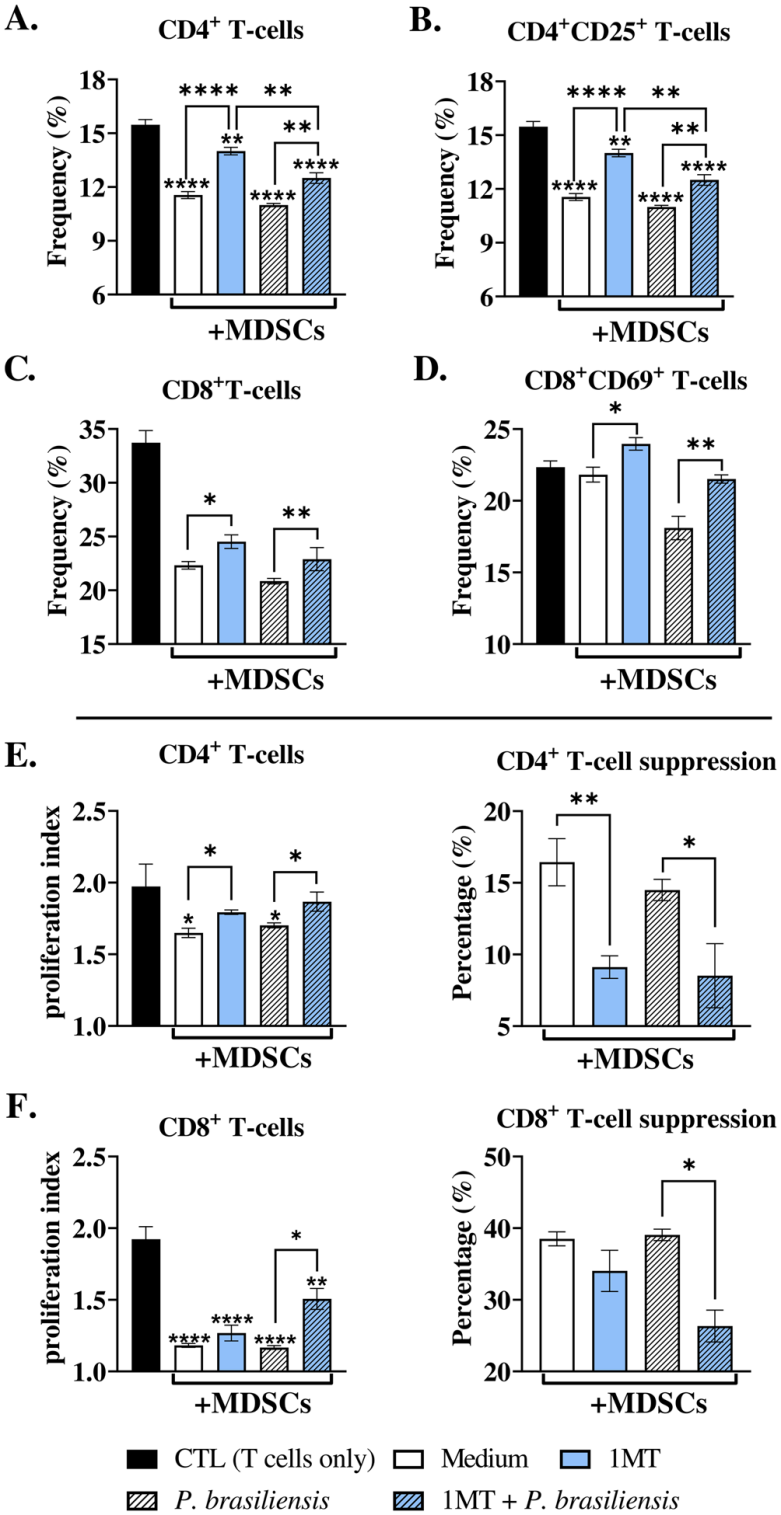
Suppression of T-cell proliferation by myeloid-derived suppressor cells (MDSCs). To evaluate the influence of the IDO-1 enzyme produced by MDSCs on the suppression of T-cell proliferation, in vitro generated MDSCs (2 × 10^5^ cells per well in a 96-well U-bottom plate) were treated or not with 1MT (200 μM), an IDO-1 inhibitor. MDSCs were challenged or not with *P. brasiliensis* yeasts in a ratio of 1:50 (yeast: MDSCs) and subsequently cocultured with 1 × 10^6^ CFSE-labeled T-cells. The T-cells were previously activated with 1 μg/mL of anti-CD3/CD28 antibodies. Following co-culture for four days (ratio of 1:10 MDSC: T-cells), the frequency of total and activated CD4 and CD8 T-cells were characterized by flow cytometric analysis (**A**–**D**), and the cell proliferation indices were obtained (**E** and **F**, left panels). To better evaluate the ability of MDSCs to suppress lymphoproliferation, the percentage of T-cell suppression was also assessed from proliferation indices (**E** and **F**, right panels). The data represent three independent experiments (*N* = 5 wells/group). (**CTL**) Control T-cells without contact with MDSCs. Differences between groups were analyzed by analysis of variance (ANOVA) followed by the Tukey test. Results were considered significant at *p < 0.05; **p > 0.01; ***p < 0.001, and ****p < 0.0001.

The treatment of MDSCs with 1MT reduced their suppressive capacity, particularly on activated CD4^+^ T-cells, as evidenced by high CD4^+^ T-cell frequencies in co-cultures with 1MT-treated MDSCs compared to untreated MDSCs (Fig. 1A and B). This suggests that IDO-1 produced by MDSCs is involved in lymphocytes immunosuppression. The addition of MDSCs to the culture impaired the proliferation of CD8^+^ T-lymphocytes. Slight differences were also observed in the frequency of total CD8^+^ T populations as well as in activated CD8^+^ T-lymphocytes when MDSCs were treated or not with 1MT (Fig. 1C and D). A higher proliferation index of the CD4^+^ T-lymphocytes was observed in co-culture with 1MT-treated compared with untreated controls, reflecting their elevated frequencies (Fig. 1E). Notably, the impaired suppression of CD8^+^ T-lymphocytes was only observed after the in vitro challenge of MDSCs with *P. brasiliensis* (Fig. 1F).

### IDO-1 absence reduced the ability of MDSCs to suppress T-cell proliferation

**Figure 2:**
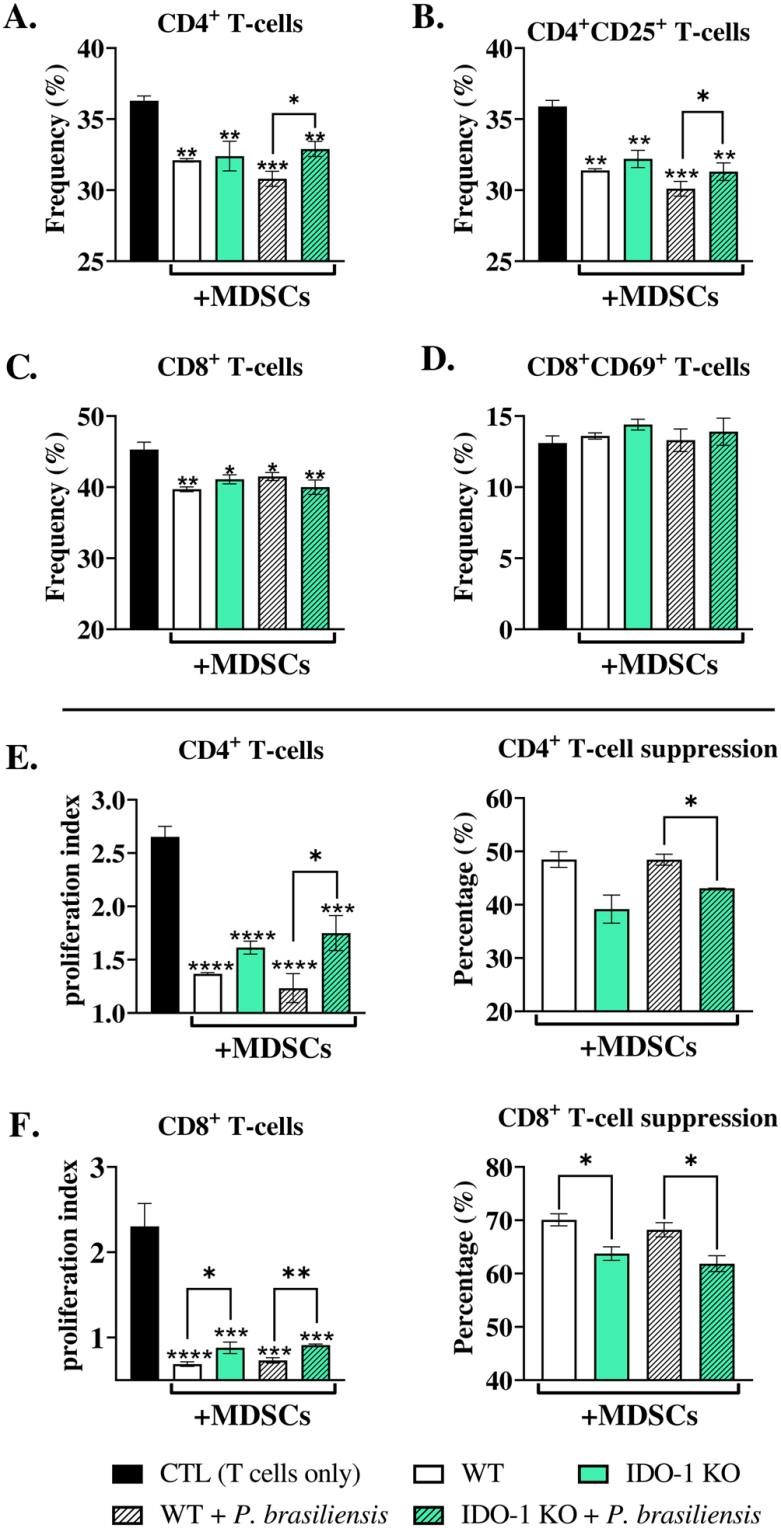
Suppression of T-cell proliferation by myeloid-derived suppressor cells (MDSCs). To evaluate the influence of IDO-1 production by MDSCs in suppressing T-cell proliferation, in vitro generated MDSCs were obtained from both wild-type (WT) and IDO-1 knockout (KO) mice. Subsequently, these MDSCs were challenged with *P. brasiliensis* yeasts at a rate of 1:50 (yeast: MDSCs) and cocultured with 1 × 10^6^ CFSE-labeled T-cells per well in a 96-well U-bottom plate. T-cells were activated previously with 1 μg/mL of anti-CD3/CD28 antibodies. Following four days of coculture (ratio of 1:10 MDSC: T-cells), the frequency of total and activated CD4 and CD8 T-cells were characterized by flow cytometric analysis (**A**–**D**). The cell proliferation indices were obtained (**E** and **F**, left panels). To evaluate the ability of MDSCs to suppress lymphoproliferation, the percentage of T-cell suppression was calculated from proliferation indices (**E** and **F**, right panels). The control T-cells (CTL) were cultured without contact with MDSCs. The data represent three independent experiments (*N* = 5 wells/group). Differences between treatments were analyzed by analysis of variance (ANOVA) followed by the Tukey test. Results were considered significant at *p < 0.05; **p > 0.01; ***p < 0.001, and ****p < 0.0001.

**Figure 3:**
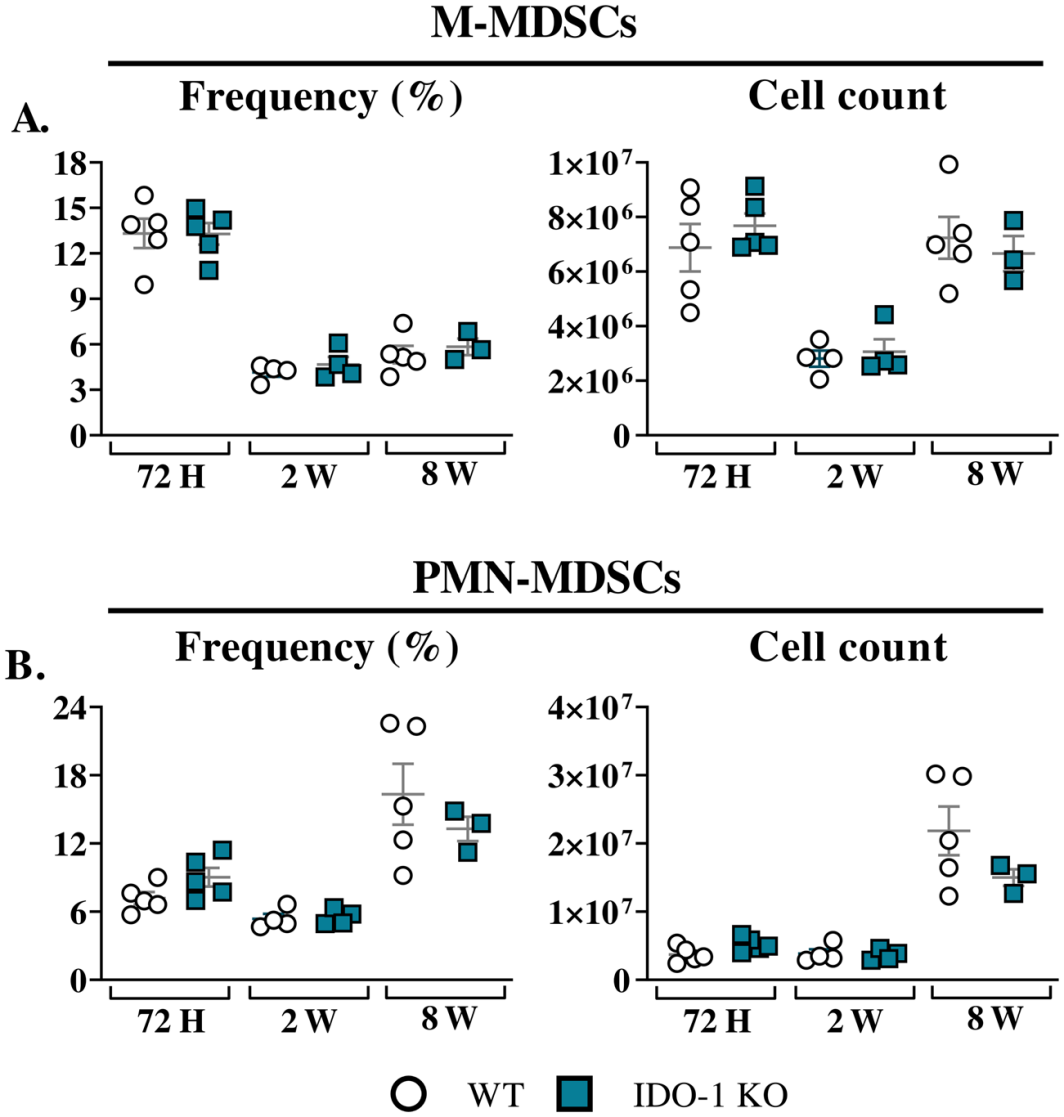
MDSCs recruitment after *P. brasiliensis* infection. C57BL/6 wild-type (WT) and IDO-1 KO mice were infected intratracheally with 1 × 10^6^ *P. brasiliensis* yeasts. Lungs were collected 72 h, two weeks, and eight weeks post infection. Specific antibodies conjugated to fluorochromes, as shown in Suppl. Fig. 1, were used to characterize MDSC subpopulations. The frequency and total cell count of lung-infiltrating M-MDSCs (**A**) and PMN-MDSCs (**B**) 72 h, two weeks, and eight weeks post infection were determined by comparing WT with IDO-1 KO. The data represent three independent experiments with 3–5 mice each. For comparisons between two groups, the mean ± SEM was obtained and analyzed by the unpaired Student’s *t*-test. Differences were considered significant at *p* < 0.05.

IDO-1-inhibited-MDSCs were generated in vitro from both WT and IDO-1KO mice and co-cultured with activated T-cells. Our findings indicate that the frequencies of CD4^+^ T and CD4^+^ CD25^+^ T-lymphocytes were higher after co-culture with IDO-1-deficient MDSCs in the presence of *P. brasiliensis* than with WT-MDSCs (Fig. 2A and B). However, the absence of IDO-1 in MDSCs did not affect the frequency of CD8^+^ T-cells (Fig. 2C), and no differences were observed in CD8^+^ T-activated cells (Fig. 2D). The analysis of T-cell proliferation revealed higher proliferation indices of CD4^+^ T-cells co-cultured with IDO-1-deficient MDSCs previously challenged with *P. brasiliensis* yeasts than those of T-cells co-cultured with WT-MDSCs, indicating an impaired suppressive capacity of IDO-1-deficient MDSCs (Fig. 2E and F). Collectively, our data demonstrate the contribution of IDO-1 to the adequate suppression of T-cell proliferation by MDSCs. Given that IDO-1 inhibition has previously been associated with decreased infiltration and activity of MDSCs in human melanoma tumors^19^, we investigated the role of IDO-1 in MDSC recruitment to the lungs of *P. brasiliensis*-infected WT and IDO-1KO mice. Our results showed that IDO-1 absence did not affect the frequency and number of lung-infiltrating MDSCs (Fig. 3A and B).

### Anti-Dectin-1 treatment or Dectin-1 absence reduced the production of IDO-1 by BM-MDSCs in vitro

**Figure 4:**
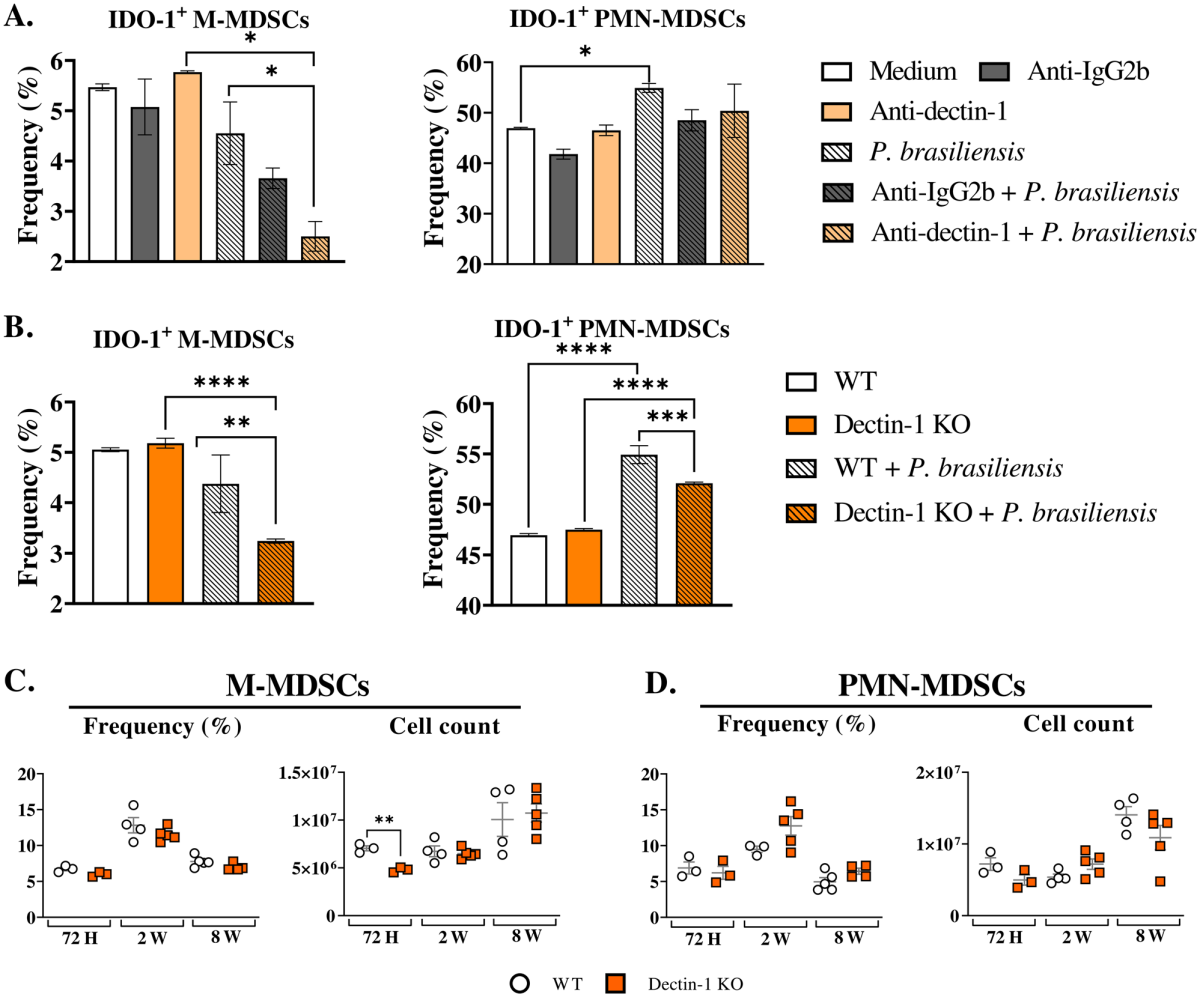
The role of Dectin-1 signaling in IDO-1 production by MDSCs. MDSCs were generated in vitro from C57BL/6 WT mice and cultured in 96-well plates (2 × 10^5^ per well). The cells were then either challenged with *P. brasiliensis* yeasts at a 1:25 ratio (yeast: MDSCs) overnight or not. Additionally, some wells were treated with 10 µg/mL of anti-Dectin-1 or anti-IgG2b for 2 h before the fungal challenge. The same fungal challenge was used to generate MDSCs from WT and Dectin-1 KO mice. (**A**) The frequencies of IDO-1^+^ M- and PMN-MDSCs following treatment with anti-Dectin-1 or control IgG2b. (**B**) The frequencies of IDO-1^+^ M- and PMN-MDSCs generated in vitro from WT and Dectin-1 KO mice. Differences between groups were analyzed by analysis of variance (ANOVA) followed by the Tukey test. The data represent three independent experiments (*N* = 5 wells/treatment). Results were considered significant at *p < 0.05; **p > 0.01; ***p < 0.001, and ****p < 0.0001. (**C** and **D**) C57BL/6 wild-type (WT) and Dectin-1 KO mice were infected intratracheally with 1 × 10^6^ *P. brasiliensis* yeasts. Lungs were collected 72 h, two weeks, and eight weeks post infection. Specific antibodies conjugated to fluorochromes, as shown in Suppl. Fig. 1, were used to characterize MDSC subpopulations. The frequency and total cell count of lung-infiltrating M-MDSCs (**C**) and PMN-MDSCs (**D**) 72 h, two weeks, and eight weeks post infection were determined by comparing WT with Dectin-1 KO. The data represent three independent experiments with 3–5 mice each. For comparisons between two groups, the mean ± SEM was obtained and analyzed by the unpaired Student’s *t*-test. Differences were considered significant at *p* < 0.05.**p > 0.01.

Previous research revealed that β-glucan (a Dectin-1 ligand) from *Saccharomyces cerevisiae* induces the expression of the tolerogenic enzyme IDO-1 in BM-derived DCs and spleen cells^20^. Given this information, along with the established role of Dectin-1 in MDSC expansion during *A. fumigatus* and *C. albicans* infections^15^, we aimed to investigate the role of Dectin-1 in the expression of IDO-1 by MDSCs during a *P. brasiliensis* infection. The IDO-1 expression was decreased by anti-Dectin-1 treatment after yeast challenge in M-MDSCs but not in PMN-MDSCs (Fig. 4A). It is important to mention that IDO-1 production was increased in untreated PMN-MDSCs after challenge with *P. brasiliensis* yeasts. Such an increase was not observed when cells were previously treated with anti-Dectin-1, suggesting an impairment of IDO-1 production in PMN-MDSCs after Dectin-1 receptor blockade followed by the fungal challenge (Fig. 4A). Besides, M-MDSCs derived from Dectin-1KO animals showed lower production of IDO-1 after challenge with *P. brasiliensis* yeasts when compared with the IDO-1 production by MDSCs from WT mice (Fig. 4B), thus confirming the results obtained with the use of the anti-Dectin-1 monoclonal antibody. Notably, the frequency of IDO-1-expressing PMN-MDSCs increased after *P. brasiliensis* challenge in WT cells but not in WT cells in the absence of fungi. The absence of Dectin-1 receptor resulted in a reduced frequency of PMN-MDSC expressing IDO-1 after fungal challenge, indicating compromised IDO-1 production by PMN-MDSCs from Dectin-1KO, as suggested by the anti-Dectin-1 inhibition with a monoclonal antibody.

### Dectin-1 absence decreased the expression of IDO-1 by lung-infiltrating M-MDSCs after *P. brasiliensis* infection

**Figure 5:**
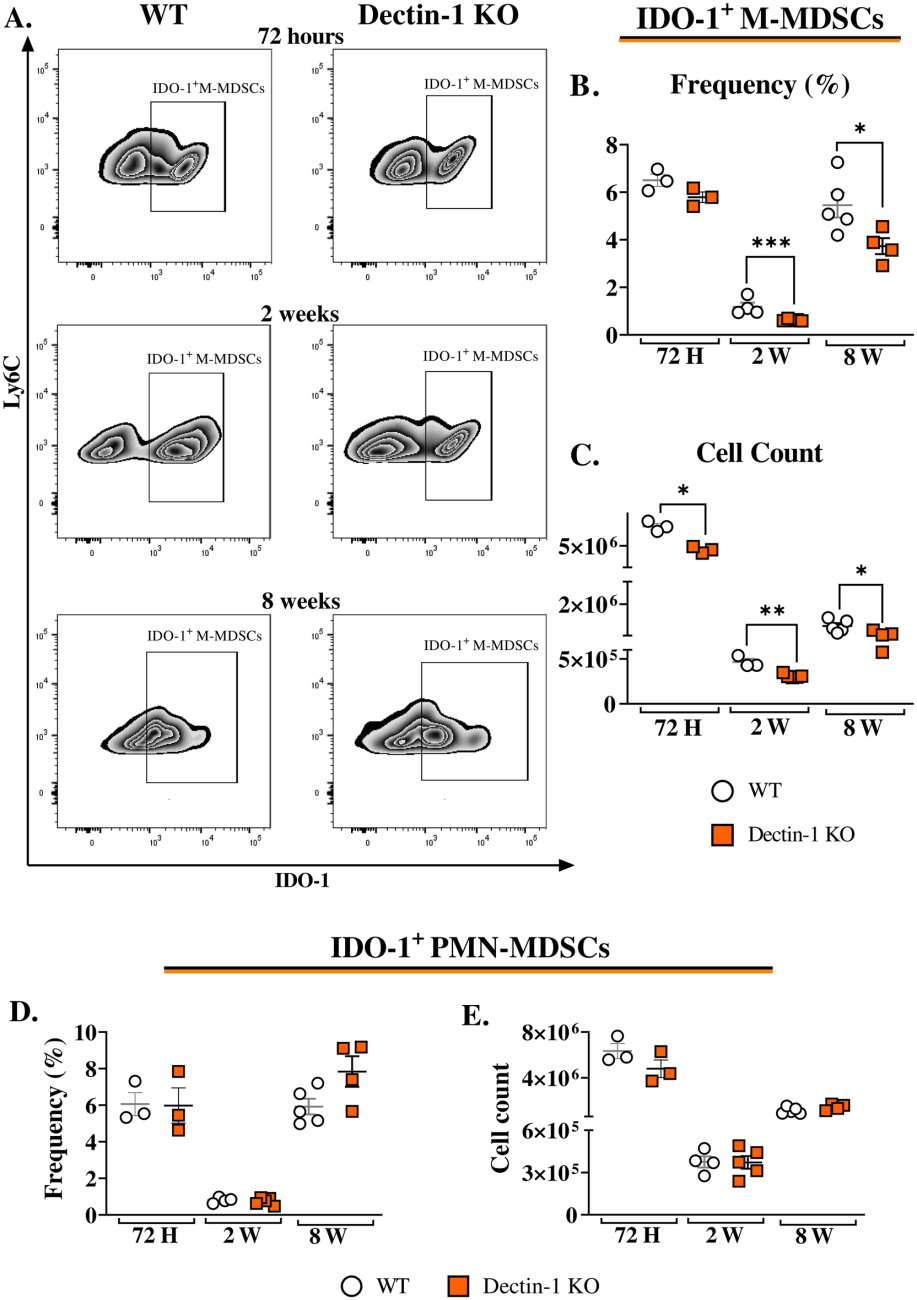
IDO-1 expression by lung-infiltrating MDSCs from WT and Dectin-1 KO mice. To verify whether Dectin-1 signaling plays a role in IDO-1 production by lung-infiltrating MDSCs, WT and Dectin-1 KO mice were infected intratracheally with 1 × 10^6^ *P. brasiliensis* yeasts, and lungs were obtained 72 h, two weeks, and eight weeks post infection. IDO-1 expression was then evaluated in M-MDSCs (**A**–**C**) and in PMN-MDSCs (**D** and **E**) through flow cytometry analysis. The displayed frequencies in smooth FACS graphs correspond to IDO-1^+^ M-MDSCs previously gated as Live/Dead- > CD45^+^ CD11b^+^  >  > Ly6G-Ly6C^high^. The data represent three independent experiments with 3–5 mice per group. For comparisons between two groups, the mean ± SEM was obtained and analyzed by the unpaired Student’s *t*-test. Differences were considered significant at *p < 0.05; **p > 0.01 ***, and *p* < 0.001.

We conducted experiments to investigate the impact of Dectin-1 on MDSC recruitment and IDO-1 expression in vivo. While previous studies have shown that Dectin-1 signaling is involved in IL-6 production^32^, which is crucial for generating and recruiting MDSCs^44^, our study reveals a reduction in the number of M-MDSCs in the lungs of Dectin-1KO mice only 72 h post infection. However, the absence of Dectin-1 did not affect the frequency of lung-infiltrating MDSCs after two and eight weeks of infection (Fig. 4C and D). Next, we evaluated whether Dectin-1 was involved in the expression of IDO-1 using WT and Dectin-1KO mice. Reduced numbers and frequencies of IDO-1^+^ M-MDSCs were observed in the lungs of Dectin-1KO mice at weeks two and eight post infection compared to WT controls (Fig. 5A, B, and C). These findings are in agreement with those obtained in vitro using anti-Dectin-1 antibodies and MDSCs from Dectin-1KO mice. Notably, unlike M-MDSCs, no differences in the frequency and number of IDO-1^+^ PMN-MDSCs were observed between the two evaluated groups (Fig. 5D and E).

### Anti-TLR2 treatment or TLR2 absence reduced IDO-1 expression by BM-MDSCs in vitro

**Figure 6:**
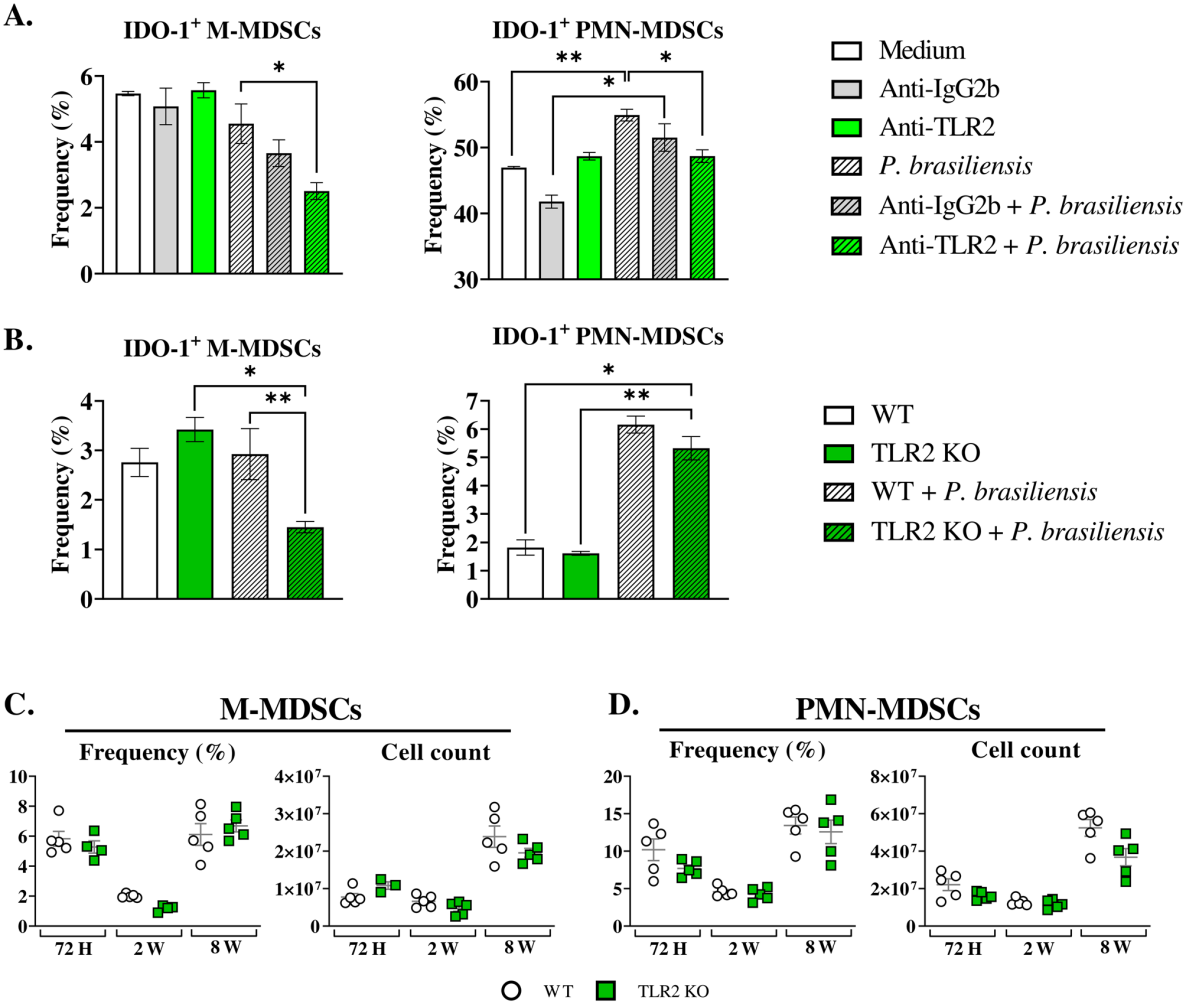
The role of TLR2 signaling in MDSCs recruitment and in IDO-1 production by MDSCs. MDSCs were generated in vitro from C57BL/6 WT mice and cultured in 96-well plates (2 × 10^5^ per well). Subsequently, the cells were either challenged or not with *P. brasiliensis* yeasts at a 1:25 ratio (yeast: MDSCs), followed by overnight incubation. Additionally, MDSCs were treated or not with 10 µg/mL of anti-TLR2 or anti-IgG2b for 2 h before the fungal challenge (**A**). In other experiments, MDSCs were generated from both WT and TLR2 KO mice and challenged with fungal yeasts as described above (**B**). (**C** and **D**) WT and TLR2 KO mice were infected intratracheally with 1 × 10^6^ *P. brasiliensis* yeasts. Lungs were collected 72 h, two weeks, and eight weeks post infection. Specific antibodies conjugated to fluorochromes, as shown in Suppl. Fig. 1, were used to characterize MDSC subpopulations. The frequency and total cell count of lung-infiltrating M-MDSCs (**C**) and PMN-MDSCs (**D**) 72 h, two weeks, and eight weeks post infection were determined by comparing WT with TLR2 KO. The data represent three independent experiments with 3–6 mice each. For comparisons between two groups, the mean ± SEM was obtained and analyzed by the unpaired Student’s *t*-test. Differences were considered significant at *p* < 0.05.

In addition to Dectin-1, TLR2, another PPR, has been shown to affect IDO-1 production. For instance, a study has shown that fractions from *Mycobacterium leprae* modulated IDO-1 production by monocyte-derived DCs in a TLR2-dependent manner^21^. Considering this and prior research indicating a role for TLR2 in the activity of MDSCs^26,27^, we aimed to investigate the impact of TLR2 signaling on IDO-1 expression by MDSCs challenged with *P. brasiliensis* yeasts in vitro. Anti-TLR2 treatment resulted in reduced IDO-1 production by M-MDSCs after *P. brasiliensis* challenge compared to untreated cells. No differences were observed between anti-TLR2 treated MDSCs and controls in the absence of the fungus. Regarding PMN-MDSCs, there was an increase in the frequency of PMN-MDSC-IDO-1^+^ in untreated cells challenged with the fungus. However, when the MDSCs were treated with anti-TLR2 followed by a fungal challenge, the PMN-MDSCs failed to increase IDO-1 production (Fig. 6A). Our results suggest a significant role for TLR2 signaling in regulating IDO-1 production by MDSCs during the *P. brasiliensis*-yeast challenge. Furthermore, M-MDSCs derived from TLR2KO animals showed impaired production of IDO-1 after challenge with *P. brasiliensis* yeasts, compared to MDSCs from WT mice, but no changes in IDO-1 production by PMN-MDSCs in TLR2KO cells were observed (Fig. 6B).

### TLR2 absence affected the expression of IDO-1 by lung-infiltrating M- and PMN-MDSCs after *P. brasiliensis* infection

**Figure 7:**
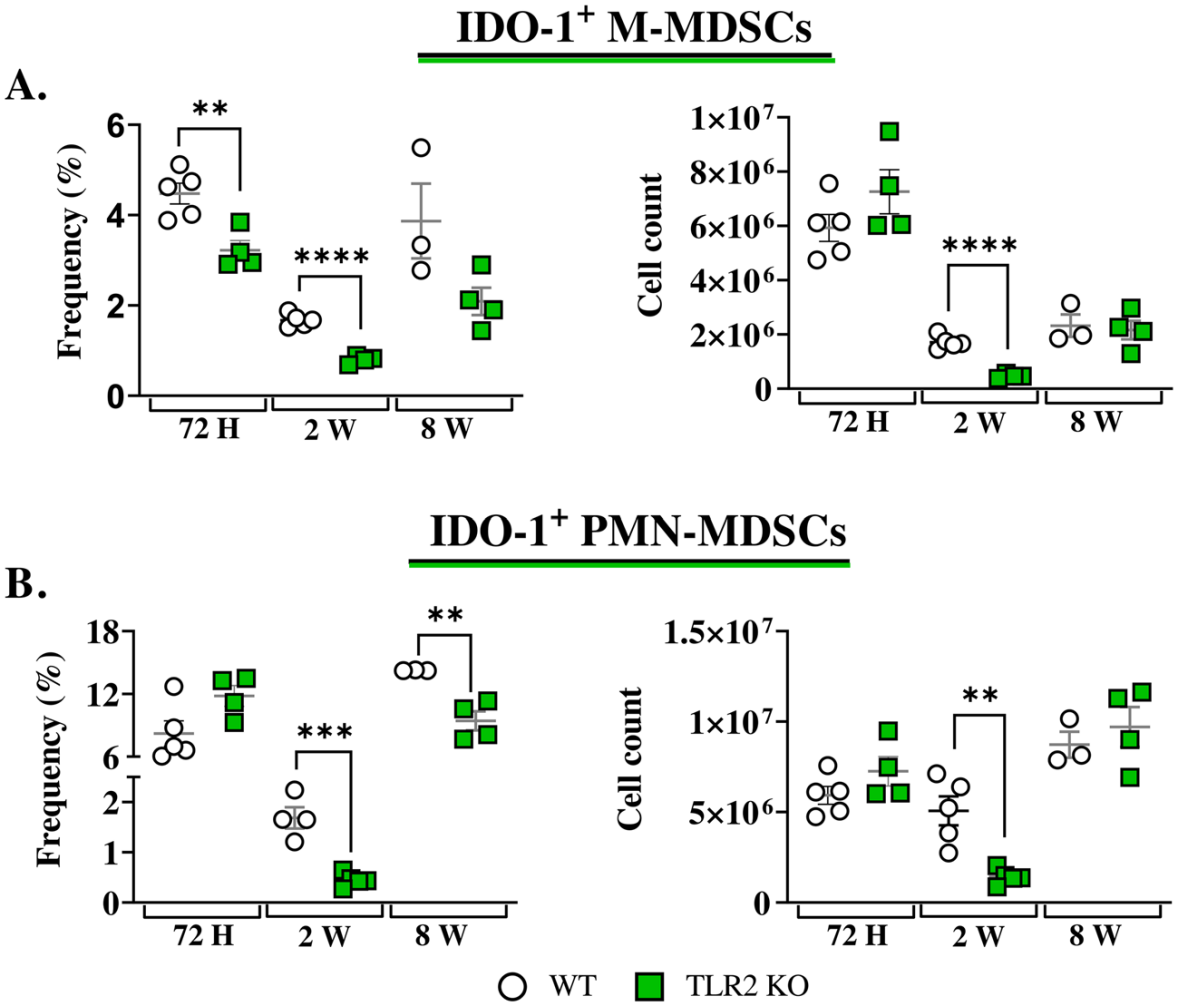
The role of TLR2 signaling in IDO-1 production by MDSCs in vivo. To verify whether TLR2 signaling plays a role in IDO-1 production by lung infiltrating MDSCs, WT and TLR2 KO mice were infected intratracheally with 1 × 10^6^ *P. brasiliensis* yeasts, and lungs were collected 72 h, two weeks, and eight weeks post infection. The expression of IDO-1 by M-MDSCs (**A**) and PMN-MDSCs (**B**) was assessed. Differences between treatments were analyzed by analysis of variance (ANOVA) followed by the Tukey test. The data presented in this figure were obtained from three independent in vivo experiments with 3–5 mice per group. For comparisons between two groups, the mean ± SEM was obtained and analyzed by the unpaired Student’s *t*-test. Differences were considered significant at *p < 0.05; **p > 0.01 ***p < 0.001, and ****p < 0.0001.

TLR2 absence did not alter the frequency or the number of lung-infiltrating MDSCs after infection when compared to WT controls (Fig. 6C and D). However, when the expression of IDO-1 was analyzed, a reduced frequency of M-MDSCs expressing IDO-1 was detected 72 h and two weeks post-*P. brasiliensis* infection. Also, a decreased number of IDO-1-producing M-MDSCs was observed after two weeks of infection (Fig. 7A). TLR2KO mice showed a reduced frequency of lung-infiltrating PMN-MDSCs expressing IDO-1 at two- and eight-weeks post infection, along with a reduced number of these cells after 2 weeks of infection, compared to wild-type controls (Fig. 7B).

### Diminished IDO-1 expression by BM-MDSCs in response to in vitro anti-TLR4 treatment and TLR4 absence

**Figure 8:**
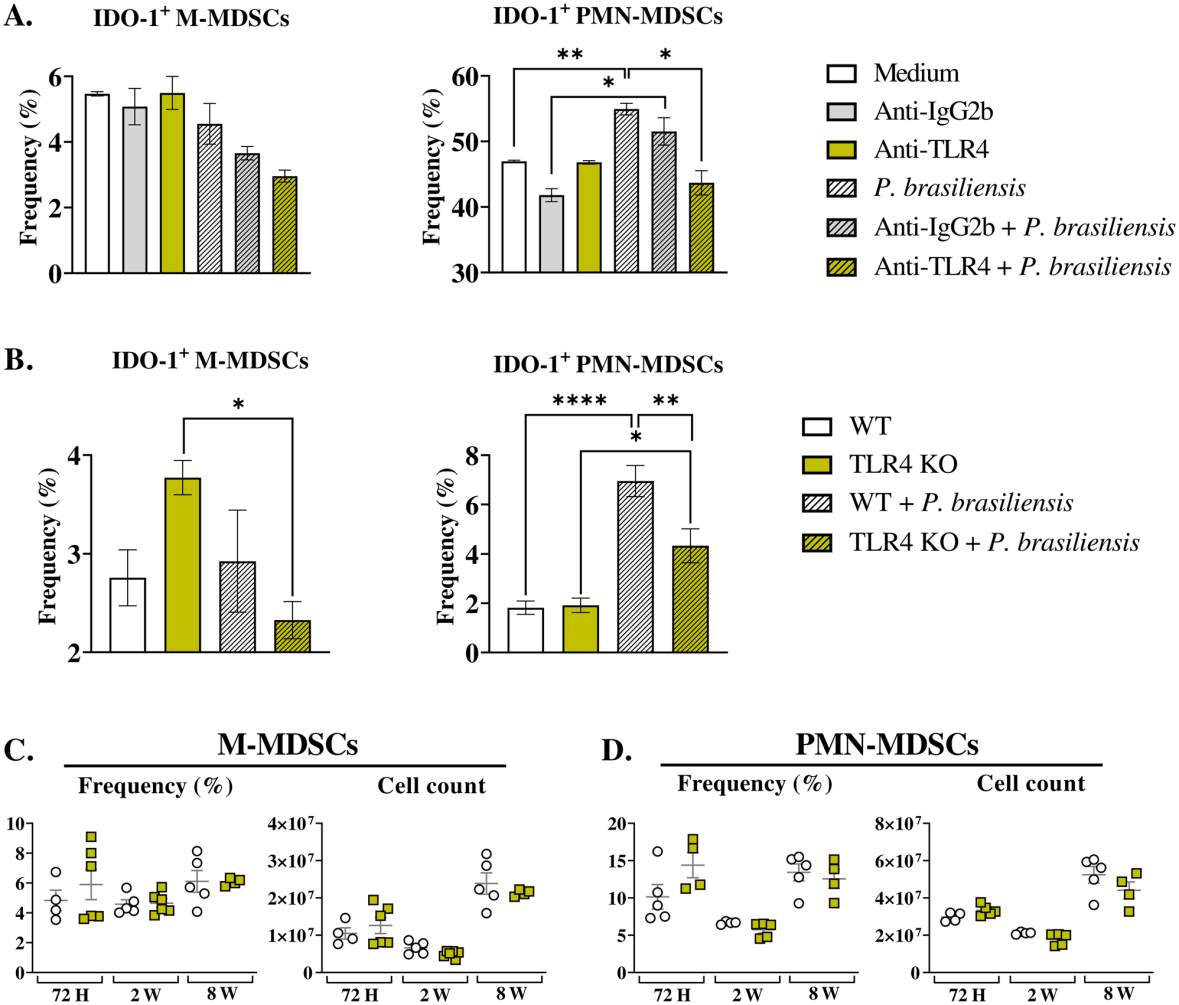
The role of TLR4 signaling in MDSCs recruitment and in IDO-1 production by MDSCs. To evaluate whether TLR4 signaling plays a role in IDO-1 production by MDSCs, MDSCs were generated in vitro from C57BL/6 WT mice and cultured in 96-well plates (2 × 10^5^ per well). The cells were subsequently challenged with *P. brasiliensis* yeasts at a 1:25 ratio (yeast: MDSCs) overnight or not. MDSCs were treated or not with 10 µg/mL of anti-TLR4 or IgG2b for 2 h before the fungal challenge (**A**). In other experiments, MDSCs were generated from WT and TLR4 KO mice, following the aforementioned fungal challenge (**B**). (**C** and **D**) WT and TLR4 KO mice were infected intratracheally with 1 × 10^6^ *P. brasiliensis* yeasts. Lungs were collected 72 h, two weeks, and eight weeks post infection. Specific antibodies conjugated to fluorochromes, as shown in Suppl. Fig. 1, were used to characterize MDSC subpopulations. The frequency and total cell count of lung-infiltrating M-MDSCs (**C**) and PMN-MDSCs (**D**) 72 h, two weeks, and eight weeks post infection were determined by comparing WT with TLR4 KO. The data represent three independent experiments with 3–6 mice each. For comparisons between two groups, the mean ± SEM was obtained and analyzed by the unpaired Student’s *t*-test. D.

TLR4, an innate immune receptor, has an impact on both IDO-1 expression^22^, and MDSC activity^25^. In this context, we investigated the contribution of TLR4 to IDO-1 expression by MDSCs in response to a *P. brasiliensis*-yeast challenge in vitro. Anti-TLR4 treatment did not affect IDO-1 production by M-MDSCs. However, in PMN-MDSCs, IDO-1 expression increased after fungal challenge, but this increase was absent in cells that received anti-TLR4 treatment (Fig. 8A). These data suggest that TLR4 signaling is involved in IDO-1 expression by PMN-MDSCs. In further assays, M-MDSCs from TLR4KO mice produced less IDO-1 after challenge with *P. brasiliensis* yeasts, while no such reduction was observed in MDSCs from WT mice. In addition, PMN-MDSCs deficient in TLR4 also displayed impaired IDO-1 production (Fig. 8B), consistent with the results obtained with TLR4-blocked MDSC.

### TLR4 deficiency reduces IDO-1 expression by lung-infiltrating MDSCs in response to *P. brasiliensis* infection

**Figure 9:**
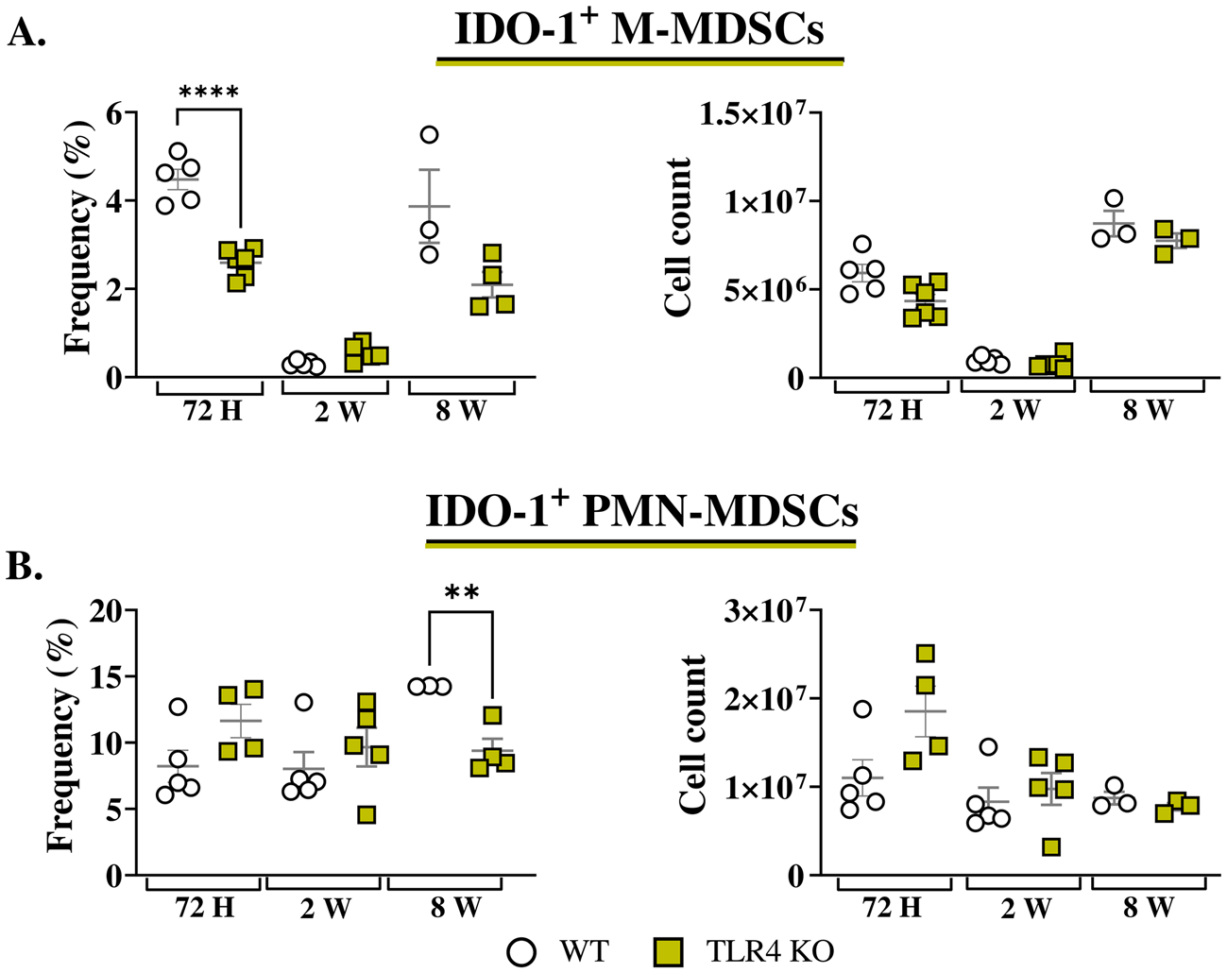
To verify whether TLR4 signaling plays a role in IDO-1 production by lung infiltrating MDSCs, WT and TLR4 KO mice were infected intratracheally with 1 × 10^6^ *P. brasiliensis* yeasts. Lungs were collected 72 h, two weeks, and eight weeks post infection. The expression of IDO-1 by M-MDSCs (**A**) and PMN-MDSCs (**B**) in the lungs was assessed. The data presented in this figure were obtained from three independent in vivo experiments with 3–6 mice per group. Differences between treatments were analyzed by analysis of variance (ANOVA) followed by the Tukey test. For comparisons between two groups, the mean ± SEM was obtained and analyzed by the unpaired Student’s *t*-test. Differences were considered significant at **, *p* < 0.001, and ****p < 0.0001.

TLR4 deficiency did not alter the frequency or number of lung-infiltrating MDSCs post infection, compared to WT controls (Fig. 8C and D). However, we did observe a decreased frequency of IDO-1^+^ M-MDSCs at 72 h post infection in TLR4-deficient mice compared to WT controls (Fig. 9A). Additionally, we detected a reduced frequency of PMN-MDSCs expressing IDO-1 in TLR4KO mice at eight weeks post infection (Fig. 9B).

## Discussion

Despite a lack of studies investigating the role of IDO-1 production by MDSCs in microbial infections, recent research has shown that other cell populations have a remarkable role in this regard. For instance, we have previously demonstrated the immunosuppressive activity of plasmacytoid dendritic cells (pDCs) via an IDO-1-dependent mechanism. The depletion of pDC-IDO-1^+^ resulted in the most severe manifestation of PCM^45^. Here, we showed that the inhibition of IDO-1 by 1MT in MDSCs co-cultured with lymphocytes resulted in a higher rate of T-cell proliferation compared to untreated MDSCs. These findings suggest that, despite the activity of other immunosuppressive mechanisms in MDSCs during *P. brasiliensis* infection^17^, IDO-1 contributes to the suppressive activity of MDSCs. Interestingly, IDO-1 expression did not enhance MDSC differentiation or influx towards the lungs of *P. brasiliensis*-infected mice. Nonetheless, our data support the previously observed reduced suppressive activity of MDSCs after IDO-1 inhibition in cancer^19,46–48^.

In autoimmune and infectious diseases, it has been demonstrated that PRRs, such as Dectin-1 and TLRs, can influence IDO-1-dependent or IDO-1-independent mechanisms of MDSC^15,23,24,49^. Rieber et al.^15^ showed that the activation of the Dectin-1/CARD9 pathway can stimulate the suppressive activity of PMN-MDSCs in the immune response against the pathogenic fungi *A. fumigatus* and *C. albicans*^15,16^. Here, we demonstrated that Dectin-1 is an important receptor for the adequate expression of IDO-1 by MDSCs in PCM. Thus, the lower number of IDO-1-producing M-MDSCs in the lungs of Dectin-1KO animals may be attributed to the pulmonary microenvironment. Furthermore, the C-type lectin-like receptor-2d, which recognizes glucuronoxylomannan, an abundant polysaccharide component of *Cryptococcus neoformans*, is fundamental in the recruitment of PMN-MDSCs in mice and patients with cryptococcosis^50^. These studies highlight the promising role of C-type lectin receptors expressed by MDSCs as immunotherapeutic targets in the treatment of fungal diseases.

The role of TLR signaling in MDSCs has been studied in several infectious diseases^24,26,27,49,51,52^. However, the role of TLR2 signaling in IDO-1 production by MDSCs, previously demonstrated in other cell populations, has not been thoroughly investigated. In *Mycobacterium leprae* infection, a decrease in IDO-1 activity was observed after TLR2 neutralization by monocyte-derived DCs^21^. Our results showed decreased IDO-1 production in TLR2-inhibited MDSCs after *P. brasiliensis* challenge. The reduction of IDO-1 expression by MDSCs obtained from *P. brasiliensis*-infected TLR2KO mice is consistent with that obtained with BM-MDSCs and TLR2 neutralization. Although our data demonstrate a correlation between TLR2 signaling and IDO-1 expression, TLR2 deficiency did not influence the recruitment of lung-infiltrating MDSCs. Interestingly, TLR2 deficiency resulted in elevated IL-6 levels in the lungs of* P*. *brasiliensis-*infected mice^29^, a cytokine required for MDSC expansion and influx to the site of infection^44,53^. Such elevated IL-6 levels could compensate for a possible negative impact on MDSC recruitment due to reduced IDO-1 expression by these cells. Notably, our data are consistent with a study with *Porphyromonas gingivalis*, the etiologic agent of periodontitis, where MDSC induction was not affected by TLR2 deficiency in mice^54^.

Elevated levels of IDO-1 can activate mature Treg cells via activation of the protein kinase general control nonderepressing-2 pathway of protein synthesis inhibition. Besides, IDO-1 produced by pDCs activates Treg cells and can further convert naïve T-cells into new Treg cell subsets^45,55^. Therefore, considering that MDSCs are an important source of IDO-1^17^, the reduced expression of this tolerogenic enzyme by lung-infiltrating M- and PMN-MDSCs in TLR2-defective mice could account for the lower expansion of Treg cells as previously described^29^.

In line with our observations on TLR2, we found no differences in the recruitment of lung-infiltrating MDSCs between WT and TLR4KO *P. brasiliensis-*infected mice. These findings coincide with a previous study of *P. gingivalis* infection, where MDSC induction was not affected by TLR4 deficiency^54^. TLR4^+^ MDSCs have been sparsely identified in select infection scenarios^25,54^, yet notable discoveries have arisen in the realm of cancer and inflammatory pathologies. For instance, chronic inflammation has been associated with the recruitment and activity of MDSCs, which can enhance the malignancy of the tumor cells. Interestingly, through co-culture experiments using MDSCs and macrophages derived from WT and TLR4KO BALB/c mice, it was demonstrated that cross-talk between inflammation-induced MDSCs and LPS-activated macrophages was TLR4-dependent. As MDSCs from TLR4KO mice failed to produce IL-10 and were less effective in reducing macrophage production of IL-12, the TLR4 pathway in MDSCs was considered a promising therapeutic target for promoting anti-tumor immune responses^56^.

To the best of our knowledge, we present the first study evaluating the role of TLR4 signaling and IDO-1 production by MDSCs in fungal infection, notwithstanding intriguing outcomes documented in other cell populations. Notably, TLR4 engagement in LPS-primed DCs under endotoxin tolerance conditions elevated levels of IDO isoforms, IL-10, and programmed death ligands 1 and 2 expression, which were contingent upon IDO, upon investigating IDO's role in inducing DC phenotype and function^57^.

Loures et al.^30^ revealed that TLR4-deficient mice displayed an upsurge in Treg cells in their lungs following *P. brasiliensis* infection, unlike their TLR2-deficient counterparts^29^. However, this increase was ascribed to reduced pulmonary fungal loads, leading to a decline in the influx of inflammatory cells to the site of infection^30^. Of note, the differences in the frequency of lung-infiltrating IDO-1^+^MDSCs between WT and TLR4KO mice observed here were not accompanied by differences in the total cell numbers, which could indicate a weak effect of TLR4 signaling for IDO-1 production by MDSCs during *P. brasiliensis* infection. These findings raise the importance of further studies addressing other regulatory molecules produced by MDSCs in PCM and other infectious diseases.

It is well established that the activation of different PRRs programs DCs to induce preferential Th1, Th2, Th17, or Treg responses. In PCM, TLR2 signaling induced by *P. brasiliensis* infection promotes anti-inflammatory responses (IL-10, TGF-β) and Treg expansion^29^. Conversely, TLR4 signaling induces a pro-inflammatory response that favors the expansion of Th1 and Th17 cells^30^. The activation of Dectin-1 and NLRP3 induces the proliferation of Th1 and Th17 lymphocytes and inhibits the expansion of Treg cells^32^. However, it is well known that IDO-1 can be induced by both pro- and anti-inflammatory mechanisms mediated by IFN-γ and TGF-β. Our previous studies have shown that IDO-1 expression by pulmonary DCs can be mediated by IFN-γ, which uses the canonical pathway (p65, 50) of the transcription factor NFκB, as well as by TGF-β, which uses the non-canonical pathway (p52, RelB) of NFκB activation^45^. Thus, it is understandable how blocked PRRs with antagonistic effects, such as those mediated by TLR2, TLR4, and Dectin-1 signaling, lead to a common effect: reduced IDO-1 expression by MDSCs.

## Conclusion

Taken together, our findings demonstrate that IDO-1 expression by MDSCs is an important mechanism for controlling T-cell proliferation and is partially dependent on Dectin-1, TLR2, and TLR4 signaling in the pulmonary PCM. Understanding the regulators and signaling pathways involved in the differentiation and activation of MDSCs is essential for developing new immunotherapeutic strategies targeting MDSCs, which has become a promising tool since our previous study has demonstrated^17^ that these cell populations are a potential target for PCM control.

## Supplementary Information

Supplementary Information.

## Data Availability

The datasets generated during and/or analyzed during the current study are available from the corresponding author on reasonable request.
