# Supplementary material for: The immunosuppressive activity of myeloid-derived suppressor cells in murine Paracoccidioidomycosis relies on Indoleamine 2,3-dioxygenase activity and Dectin-1 and TLRs signaling
Source: Sci Rep. 2023 Jul 31;13:12391. doi: 10.1038/s41598-023-39262-8 (PMC10390561; doi:10.1038/s41598-023-39262-8)
Supplement: Supplementary file 1 — Supplementary Information. [file 41598_2023_39262_MOESM1_ESM.pdf]

Supplementary Information

The immunosuppressive activity of myeloid-derived suppressor cells in murine Paracoccidioidomycosis relies on Indoleamine 2,3-dioxygenase activity and Dectin-1 and TLRs signaling

Valéria de Lima Kaminski[1]; Nycolas Willian Preite[1]; Bruno Montanari Borges[1]; Bianca Vieira dos Santos[1]; Vera Lucia Garcia Calich[2]; Flávio Vieira Loures[1]

[1] Institute of Science and Technology, Federal University of São Paulo – UNIFESP, São José dos Campos, São Paulo, Brazil.

[2] Department of Immunology, Institute of Biomedical Sciences, University of São Paulo – USP, São Paulo, Brazil.

*** Corresponding Author**: Flávio Vieira Loures; e-mail: loures@unifesp.br

Authors' e-mail adresses:

Valéria de Lima Kaminski (VLK): kaminski.valeria@unifesp.br

Nycolas Willian Preite (NWP): preite@unifesp.br

Bruno Montanari Borges (BMB): bmborges@unifesp.br

Bianca Vieira dos Santos (BVS): bsantos25@unifesp.br

Vera Lúcia Garcia Calich (VLGC): vlcalich@icb.usp.br

Supplementary Figure 1

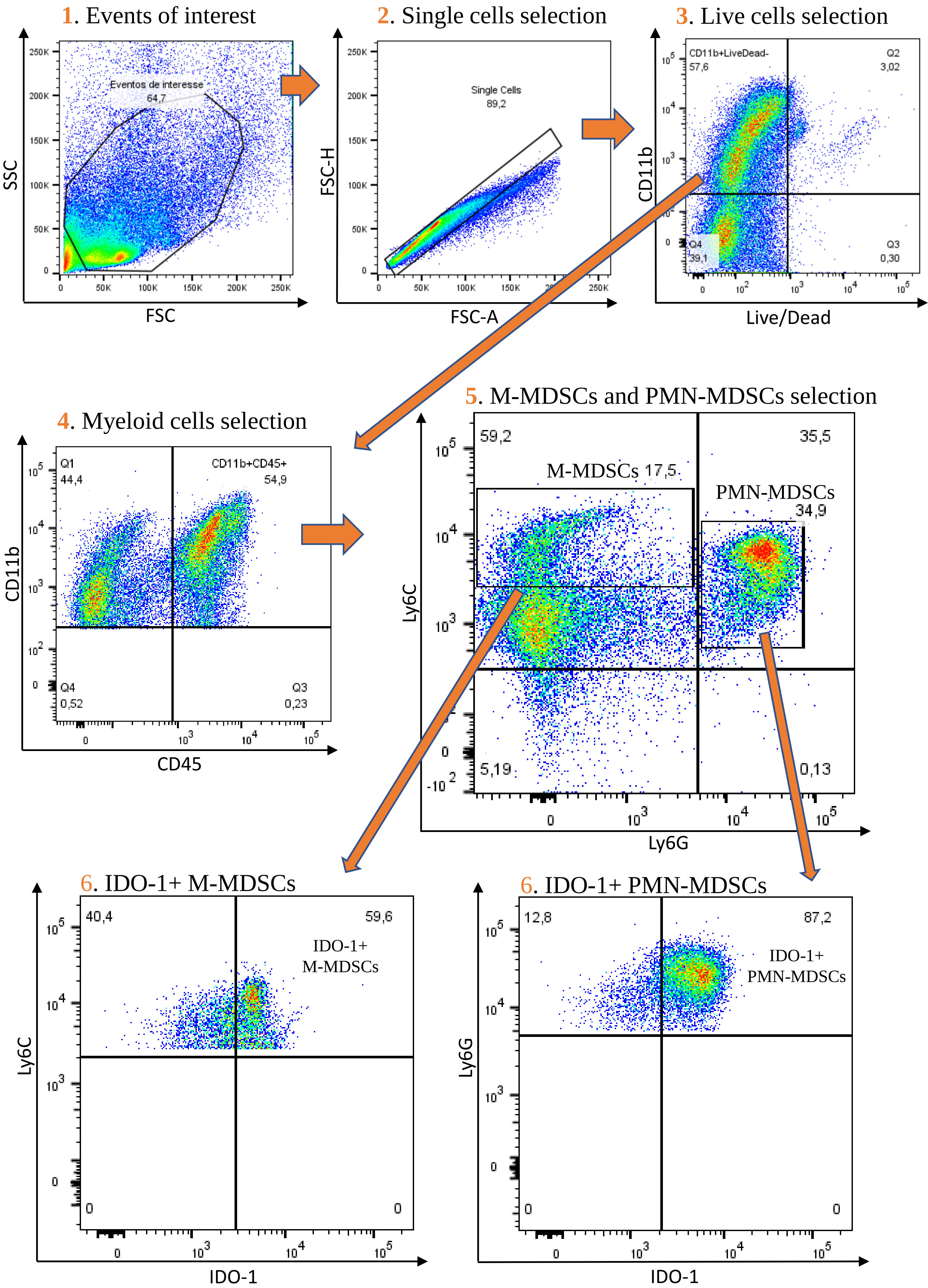

Figure Legend

Supplementary Figure 1. Immunophenotyping of MDSCs. This figure shows the gating strategy used for identifying MDSCs and IDO-1 production by these cells. Cells were gated by FSC/SSC analysis, and populations of M-MDSCs (Live/Dead- $CD45^{+}CD11b^{+}LY6G^{-}LY6C^{hi}$) and PMN-MDSCs ($CD45^{+}CD11b^{+}LY6G^{+}LY6C^{low}$) generated *in vitro* or recovered from infected lungs were assessed (**A)** and intracellularly stained with an anti-IDO-1 antibody **(B**). The gating strategy represented in this figure is from *P. brasiliensis* infected mice lungs, 72 h post-infection. Samples were run on a FACS Lyric flow cytometer, and 100,000 events were acquired. Analysis was performed using FlowJo software (Tree Star).

Supplementary Figure 2

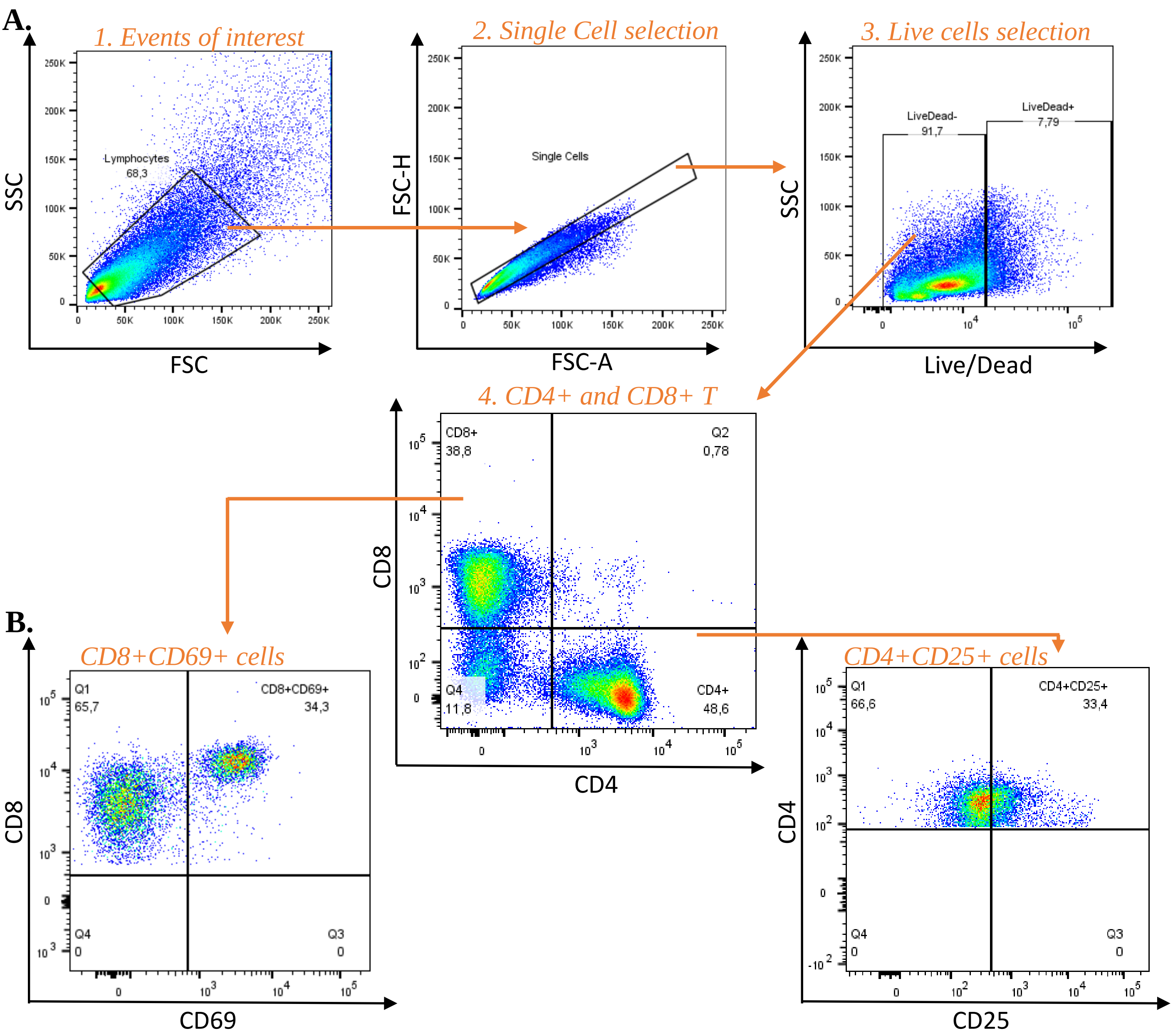

Figure Legend

Supplementary Figure 2. Magnetically sorted T-cells from naïve mice spleens were labeled with CFSE, activated with andi-CD3/CD28 and cultured for 4 days in the presence or absence of MDSCs (exposed or not to *P. brasiliensis* yeasts). One million T cells were placed by well in a 96-well U-bottom plate. T cells where then characterized as CD4+, CD8+, CD4+CD25+ and CD8+CD69+. Samples were run on a FACS Lyric flow cytometer, and 50,000 events were acquired in samples. Analysis was performed using FlowJo software (Tree Star).

Supplementary Figure 3

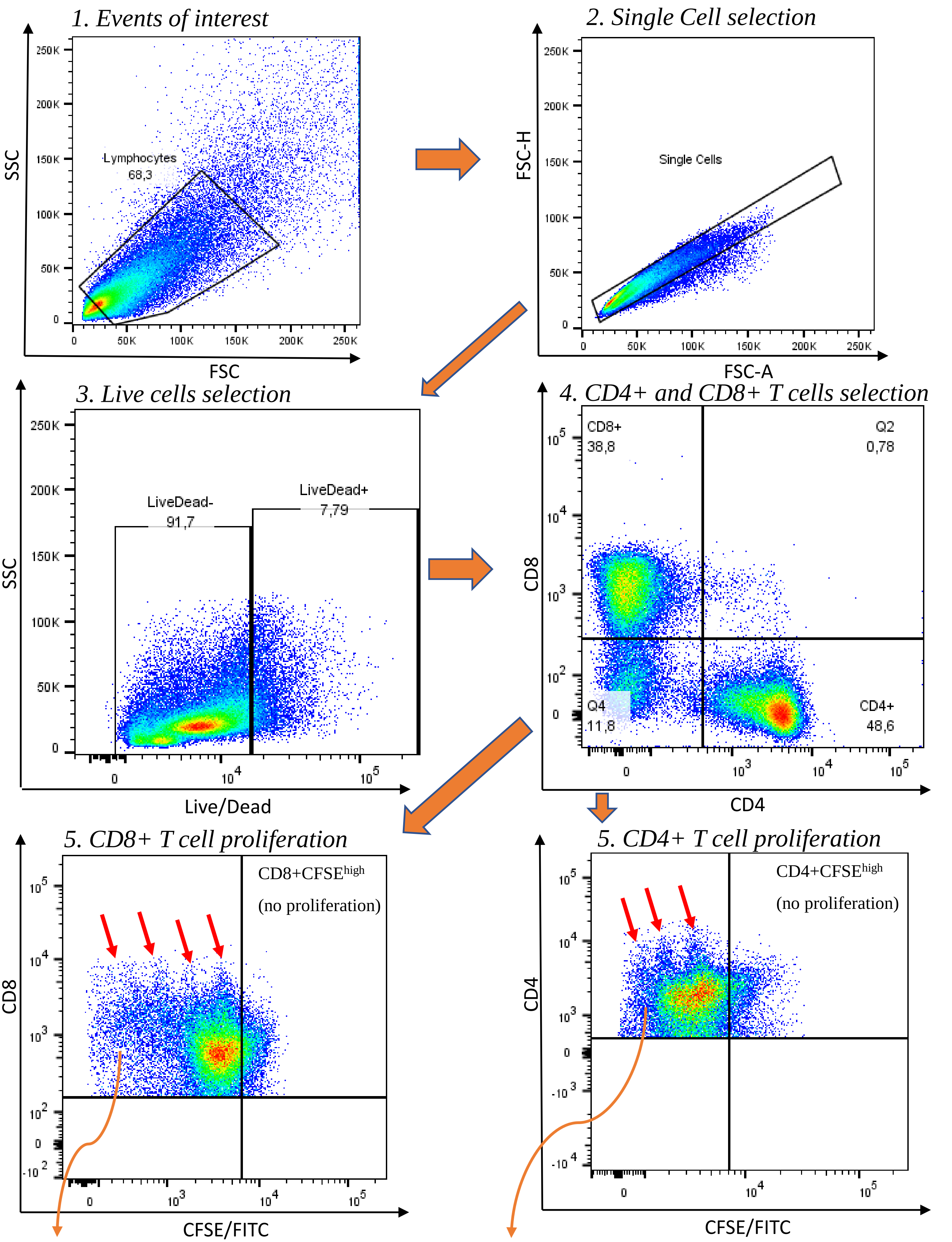

Red arrows indicate CFSE dilution. CFSE high cells were excluded from the analysis. The cells positioned in the gates where the arrows are were analyzed according to the geometric mean of the CFSE dilution of the cells. The calculation was performed according to Mannering et al. (2003), as already presented by our group in Preite et al. (2023).

Figure Legend

Supplementary Figure 3. Lymphoproliferation. Magnetically sorted T-cells from naïve mice spleens were labeled with CFSE, activated with andi-CD3/CD28 and cultured for 4 days in the presence or absence of MDSCs (exposed or not to *P. brasiliensis* yeasts). One million T cells were placed by well in a 96-well U-bottom plate. T cells where then characterized as CD4+ or CD8+ and CFSE fluorescence was assessed by the FITC channel. Samples were run on a FACS Lyric flow cytometer, and 50,000 events were acquired in samples. Analysis was performed using FlowJo software (Tree Star).
